# Contraction Band Necrosis with Dephosphorylated Connexin 43 in Rat Myocardium after Daily Cocaine Administration

**DOI:** 10.3390/ijms231911978

**Published:** 2022-10-09

**Authors:** Shuheng Wen, Kana Unuma, Takeshi Funakoshi, Toshihiko Aki, Koichi Uemura

**Affiliations:** Department of Forensic Medicine, Graduate School of Medical and Dental Sciences, Tokyo Medical and Dental University, Tokyo 113-8519, Japan

**Keywords:** cocaine, contraction band necrosis, connexin 43, adiponectin, apoptosis, mitochondria

## Abstract

Contraction band necrosis (CBN) is a common abnormality found in the myocardium of cocaine abusers, but is rarely reported in experimental models of cocaine abuse. Connexin 43 (Cx43) is essential for cardiac intercellular communication and the propagation of CBN. Under stress or injury, cardiac Cx43 is dephosphorylated, which is related to cardiomyocyte dysfunction and pathogenesis, whereas adiponectin exerts beneficial effects in the myocardium. In this study, we explore the effects of cocaine on cardiac Cx43 in vivo. Rats were administered cocaine via the tail vein at 20 mg/kg/day for 14 days, and showed widespread CBN, microfocal myocarditis and myocardial fibrosis, corresponding to a dysfunction of cardiac mitochondria under increased oxidative stress. The increase in dephosphorylated cardiac Cx43 and its negative correlation with the myocardial distribution of CBN after cocaine administration were determined. In addition, apoptosis and necroptosis, as well as increased adiponectin levels, were observed in the myocardium after cocaine exposure. Accordingly, we found altered profiles of cardiac Cx43, CBN and its negative correlation with dephosphorylated cardiac Cx43, and the possible involvement of adiponectin in the myocardium after 14 days of cocaine administration. The latter might play a protective role in the cardiotoxicity of cocaine. The current findings would be beneficial for establishing novel therapeutic strategies in cocaine-induced cardiac consequences.

## 1. Introduction

Cocaine is well-established to induce serious cardiovascular damage [1,2]. The cardiotoxicity of cocaine derives from its potent blockage of sodium channels, persistent elevation of catecholamine levels, and disruption of cellular Ca2+ homeostasis [3]. These effects result in elevated myocardial oxygen demand while simultaneously reducing myocardial oxygen supply [4,5,6]. The cocaine-induced excessive catecholamine and anoxia in the myocardium lead to an overwhelming increase in the intramyocellular Ca^2+^ concentration, which results in hypercontraction of the sarcomeres and subsequent myofibrillar rhexis, a condition known as contraction band necrosis (CBN, also referred to as coagulative myocytolysis) [7,8,9]. CBN appears as hypereosinophilic transverse bands in myocytes in isolated lesions or microscale clusters [10]. Instead of a pathognomonic feature, CBN can be observed in various conditions, including ischemic heart disease [11] and resuscitation [12]. It is considered one of the most common pathological findings in the myocardium of cocaine abusers [8]. The propagation of CBN in the myocardium is bound up with the potency of gap junctions, wherein cardiac connexin 43 (Cx43) is believed to play an essential role [13,14,15].

Cardiac Cx43 exists in myocardial gap junctions, which are primarily localized in intercalated discs [16,17]. In healthy myocardium, gap junctional Cx43 is in a phosphorylated state to enhance normal intercellular communication, which enables the exchange of electrical stimuli and synchronous myocardial contraction [18,19,20]. Sufficient evidence has been obtained from cardiac ischemia/reperfusion models to indicate the cardioprotective role of phosphorylated Cx43 [17], whereas under pathological conditions, dephosphorylated Cx43 undergoes lateral redistribution from the gap junctions and subsequent degradation, which results in the weakening of intercellular communication [21]. The dephosphorylation of cardiac Cx43 is related to cardiomyocyte apoptosis and myocardial fibrosis [22], as observed in arrhythmias, ischemic myocardial injury, and heart failure [23,24]. Similar cardiac consequences are observed under cocaine exposure, yet whether cardiac Cx43 plays a role in cocaine cardiotoxicity remains to be elucidated.

Adiponectin is a protein hormone that protects the cardiovascular system against stress and injury, as well as against numerous detrimental conditions, including atherogenesis, inflammation, and fibrosis [25,26,27]. AMP-activated protein kinase (AMPK) is a key mediator in most of these beneficial effects [28]. In addition, it has been determined that, in diabetic and septic models, adiponectin mitigates myocardial inflammation and dysfunction by regulating cardiac Cx43 expression [29,30]. However, the involvement of adiponectin in cocaine cardiotoxicity is still unknown.

Herein, we investigated the effects of cocaine on cardiac homeostasis using an in vivo model, in which rats were administered cocaine at a dose of 20 mg/kg/day via the tail vein for 14 days. We found widespread CBN and microfocal myocarditis and fibrosis in the myocardium after cocaine administration. Correspondingly, cardiac mitochondrial dysfunction and increased oxidative stress were observed. We also found an increase in dephosphorylated cardiac Cx43, and a negative correlation between CBN and dephosphorylated Cx43 in myocardial distribution after cocaine administration. Furthermore, the activation of pathways responsible for apoptosis and necroptosis, as well as the participation of adiponectin, were found in the myocardium after cocaine exposure. We believe that this is the first in vivo study revealing the alteration in the cardiac Cx43 profile and indicating the possible involvement of adiponectin in cardiomyocytes after cocaine administration. Taking advantage of the current findings, novel therapeutic strategies against cardiac consequences in cocaine addicts could be explored.

## 2. Results

### 2.1. Pathological Changes in Myocardium after 14 Days of Cocaine Administration via Tail Vein

**Figure 1:**
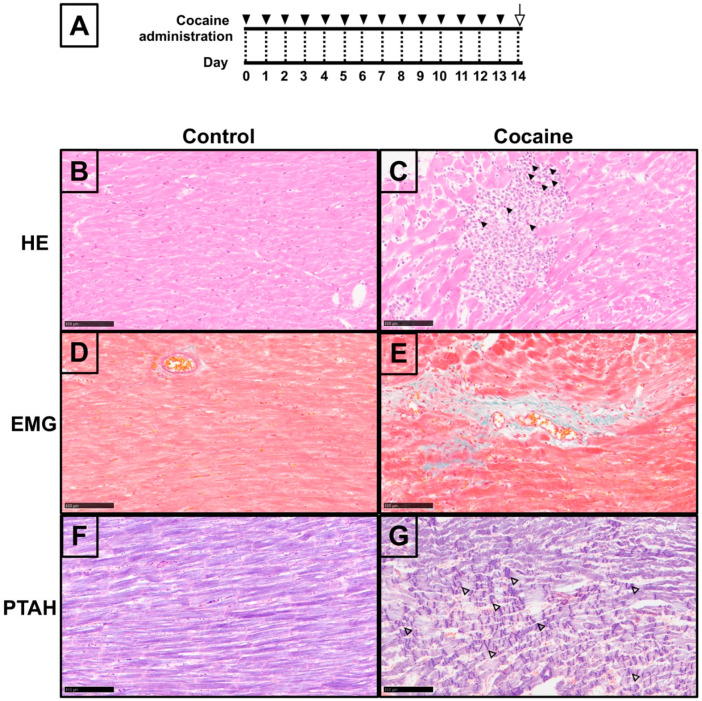
Microscopy of myocardium after 14 days of cocaine administration. (**A**) Timeline of experimental procedures. Rats were administrated with cocaine (20 mg/kg/day) and sacrificed on day 14. Filled arrowheads indicate cocaine administration. The open arrow indicates sample collection from sacrificed rats. (**B**) HE staining, (**D**) EMG staining, and (**F**) PTAH staining of the myocardium samples from the control group; (**C**) HE staining of the myocardium from the cocaine group, indicating myocarditis with eosinophil infiltration (arrowheads), focal myocytolysis, swollen cardiomyocyte nuclei, and bright myocardial cytoplasm around the myocarditis; (**E**) EMG staining of the myocardium from the cocaine group, showing perivascular fibrosis, abundant green-stained collagen fibers around the microvascular, and infiltration into the interstitium; (**G**) PTAH staining of the myocardium from the cocaine group, exhibiting broad transverse contraction band lesions (hollow arrowheads), and wavy myocardial fibers around the lesions. Scale bars = 100 μm.

Sufficient evidence from in vivo animal models has shown a relationship between cardiovascular disorders and cocaine exposure via intraperitoneal administration [31,32]. Thus, Hematoxylin-eosin (H&E) and Elastica Masson-Goldner (EMG) staining were performed in order to assess the condition of the myocardium in our cocaine abuse model, applying tail vein administration (Figure 1B–G). The H&E results revealed microfocal myocarditis in the myocardium of the cocaine group with eosinophil-dominated infiltration. Focal myocytolysis, swollen cardiomyocyte nuclei, and bright myocardial cytoplasm were found around the myocarditis regions (Figure 1B,C). EMG staining of the myocardium from the cocaine group showed scattered perivascular fibrosis, with abundant green-stained collagen fibers surrounding the microvascular and infiltration into the interstitium (Figure 1D,E). Furthermore, we observed widespread CBN-like hypereosinophilic transverse bands in myocardium from the cocaine group (Supplementary Figure S1), which we verified by Phosphotungstic Acid Hematoxylin (PTAH) staining [33]. PTAH staining verified CBN in the cocaine group by revealing extensive dense transverse contraction band lesions, accompanied by wavy myocardial fibers in the adjacent region (Figure 1F,G). Collectively, the histological findings demonstrate the pathological condition of the myocardium after 14 days of cocaine administration via the tail vein.

### 2.2. Cardiac Mitochondrial Dysfunction and Elevated Oxidative Stress in Myocardium

**Figure 2:**
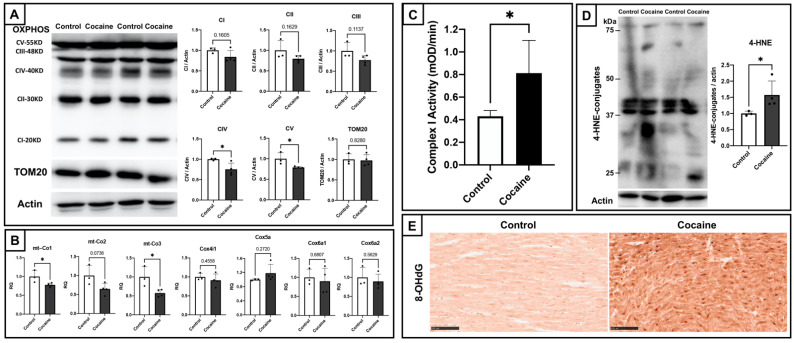
Cardiac mitochondrial dysfunction and elevated oxidative stress after 14 days of cocaine administration. (**A**) Protein expression levels of mitochondria oxidative phosphorylation subunits (OXPHOS) and translocase of outer mitochondrial membrane 20 (TOM20) in the rat’s left ventricle after cocaine administration. Levels of actin served as the internal control, CI~V, complex I~V. (**B**) Expression levels of cytochrome c oxidase subunit I (*mt-Co1*), cytochrome c oxidase subunit II (*mt-Co2*), cytochrome c oxidase subunit III (*mt-Co3*), cytochrome c oxidase subunit 4I1 (*Cox4i1*), cytochrome c oxidase subunit 5A (*Cox5a*), cytochrome c oxidase subunit 6A1 (*Cox6a1*), and cytochrome c oxidase subunit 6A2 (*Cox6a2*) after cocaine administration, as examined by qPCR. GAPDH levels served as an endogenous control. (**C**) Detection of endogenous Cox I activity in rat cardiac mitochondria isolated from myocardium samples of control group and cocaine group rats. (**D**) Protein expression of 4-hydroxy-2-nonenal (4-HNE) modified proteins in the rat’s left ventricle after cocaine administration. (**E**) Immunohistochemical analyses of 8-OHdG in the myocardium. Increased immunoreactivities to 8-OHdG in the cocaine group are shown. Scale bars = 100 μm. Levels of actin served as the internal control. Individual values are presented in black bullets. Each bar represents mean and S.D. *, *p* < 0.05.

Mitochondrial dysfunction and oxidative stress play prominent roles in the cardiotoxicity of cocaine. Therefore, we determined the effects of cocaine on cardiac mitochondria. Firstly, we examined the status of cardiac mitochondrial oxidative phosphorylation. Immunoblotting of mitochondrial oxidative phosphorylation (OXPHOS) subunit proteins and the outer mitochondrial membrane 20 (TOM20) protein revealed a general trend toward decreased expressions, with significant decreases found for the expressions of complex IV and V proteins (Figure 2A). Complex IV is believed to play a central role in mitochondrial oxidative metabolism and oxidative stress [34]. Following the detection of transcripts, regulating complex IV determined significant downregulations of cytochrome c oxidase subunit I *(MT-CO1*) and cytochrome c oxidase subunit III (*MT-CO3*) (Figure 2B). These represent an impairment of mitochondrial bioenergetic function by cocaine cardiotoxicity. In contrast, endogenous OXPHOS complex I activity assays showed significantly elevated complex I activity in the cocaine group as compared to the control group (Figure 2C). Considering that the production of intracellular oxidative stress is mainly attributed to complex I [35], and that excessive complex I activity might lead to an elevation in the generation of reactive oxygen species (ROS), we examined the oxidative stress markers 4-hydroxy-2-nonenal (4-HNE) and 8-hydroxy-2′-deoxyguanosine (8-OHdG), which found apparently elevated oxidative stress in the myocardium of the cocaine group (Figure 2D,E). These findings suggest cardiac mitochondrial dysfunction after 14 days of cocaine administration.

### 2.3. Electron Microscopic Analysis of Myocardium after 14 Days of Cocaine Administration via Tail Vein

**Figure 3:**
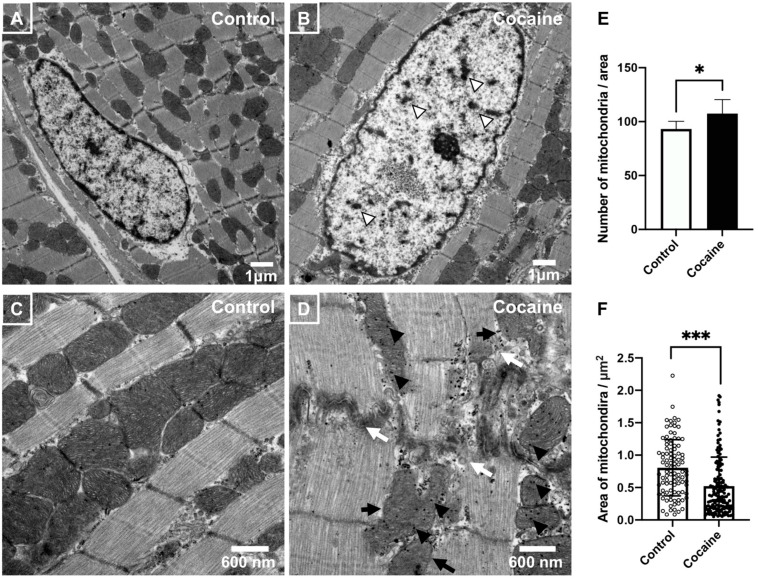
Transmission electron micrographs of left ventricle samples from the control and cocaine groups. (**A**,**B**) Nucleus of cardiomyocytes: the nuclei in the cocaine group shows lighter nucleoplasm and chromatin clumping (hollow arrowheads) as compared to the nuclei in the control group, scale bars = 1μm. (**C**) Control group: normally arranged myofibers and mitochondria, evenly sized mitochondria with intact matrix. (**D**) Cocaine group: discontinuous and ruptured myofibers (white arrows), irregularly shaped mitochondria with unclear matrix structure (black arrows) and mitochondrial densities (black arrowheads; a sign of Ca^2+^ overload), scale bars = 600 nm. (**E**) The number of mitochondria per field in control and cocaine group rats. (**F**) Mitochondrial area in control and cocaine group rats. Comparison of the distribution of the mitochondrial area in control and cocaine group rats is shown. Over 100 mitochondria were measured in each group; areas were measured in the longitudinal view of myofibrils. Each white and black bullet stands for the area of measured mitochondrion in control and cocaine group, respectively. Each bar represents the mean and S.D., *, *p* < 0.05, ***, *p* < 0.001.

Given the indications of mitochondrial damage by cocaine administration (Figure 2), transmission electron microscopy was performed for the direct observation of cardiac mitochondria. The nuclei of cardiomyocytes in the cocaine group showed lighter nucleoplasm and chromatin clumping as compared to the nuclei in the control group (Figure 3A,B). This observation is in accordance with the nuclei of cardiomyocytes under stress or injury, similarly indicating a pathological condition of the myocardium of the cocaine group. Moreover, compared to the normally arranged myofibers, as well as well-shaped and evenly sized mitochondria with intact matrices, as observed in the control group, the cocaine group presented with discontinuous and ruptured myofibers, irregularly shaped mitochondria with unclearly structured matrices, and highly dense dots within or around mitochondria, which reflects Ca^2+^ overload (Figure 3C,D). In addition, we found a significantly increased cardiac mitochondria number with a simultaneous remarkable reduction in the cardiac mitochondrial cross-sectional area, as observed for the longitude sections of myocardial fibers in the cocaine group (Figure 3E,F). In general, the disrupted mitochondrial dynamics and mitochondrial fragmentation reflect dysfunction and injure cardiac mitochondria [36]. Therefore, we conclude that these observations confirm damage to cardiac mitochondria and cardiomyocytes after 14 days of cocaine administration.

### 2.4. Dephosphorylated Cardiac Cx43 and Its Distribution in Myocardium

**Figure 4:**
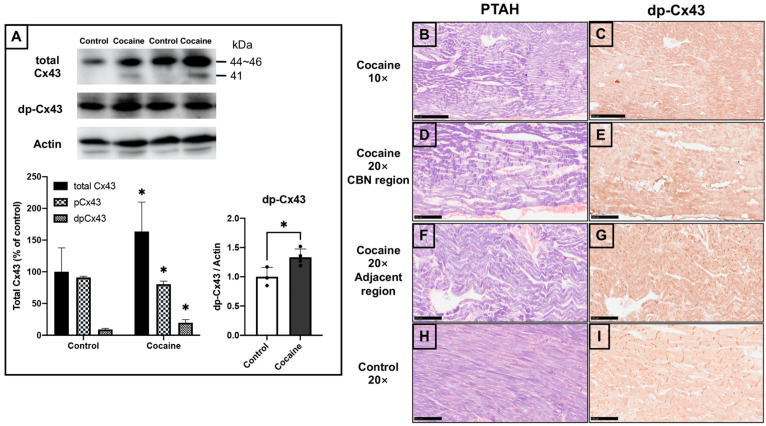
Dephosphorylated cardiac Connexin 43 and its distribution in myocardium after 14 days of cocaine administration. (**A**) The protein expressions of total Connexin43 (Cx43) and dephosphorylated Cx43 at Serine 368 (dp-Cx43) in the rat’s left ventricle after cocaine administration. Levels of actin served as the internal control. Band densities of total, phosphorylated, and dephosphorylated Cx43 in the cocaine group relative to the means of the control group. Individual values are presented in black bullets. Each bar represents mean and S.D. *, *p* < 0.05 (**B**) PTAH staining of myocardium samples from the cocaine group, scale bar = 250 μm. (**D**,**F**) PTAH staining of myocardium samples from the cocaine group, scale bar = 100 μm. (**H**) PTAH staining of myocardium samples from the control group, scale bar = 100 μm. (**C**) Immunohistochemistry of dp-Cx43 in left ventricle samples from the cocaine group, scale bar = 250 μm. (**E**,**G**) Immunohistochemistry of dp-Cx43 in left ventricle samples from the cocaine group, scale bar = 100 μm. (**I**) Immunohistochemistry of dp-Cx43 in left ventricle samples from the control group, scale bars = 250 μm.

In view of the role of Cx43 in CBN propagation and cardiac pathogenesis, we investigated Cx43 expression in the myocardium. Immunoblotting of total cardiac Cx43 showed a significantly increased Cx43 level, with a decreased percentage of phosphorylated Cx43 (44–46 kDa) and an increased percentage of dephosphorylated Cx43 (41 kDa) in the cocaine group (Figure 4A). The separate immunoblots for dephosphorylated Cx43 at serine 368 also showed an accordingly significant increase in the myocardium of the cocaine group, verifying the dephosphorylation of Cx43 after cocaine administration. This suggests an abnormal functioning of cardiac gap junctions after cocaine administration. The results of PTAH staining and immunochemistry of dephosphorylated Cx43 were compared in order to explore the association between CBN and Cx43 in the cardiotoxicity of cocaine (Figure 4B–I). Intriguingly, the region staining positive for dephosphorylated Cx43 in the histochemistry results appeared to be negatively correlated with the highly-stained region in the PTAH results in the myocardium of the cocaine group (Figure 4B,C). In the CBN region of the myocardium from the cocaine group, the staining seems to be restricted to the cytosol and nucleus, while in the areas adjacent to or absent from CBN, the dephosphorylated Cx43 staining was mostly observed at the intercalated discs (Figure 4D–G). In addition, the level of dephosphorylated Cx43 staining was increased in the cocaine group as compared to the control, which is consistent with the immunoblot results showing an increased level of dephosphorylated Cx43 (Figure 4G,I). The expression and distribution of cardiac Cx43 demonstrates the dephosphorylation of Cx43 and its negative connection with CBN in the myocardium after 14 days of cocaine administration.

### 2.5. Involvement of Apoptosis and Necroptosis in the Damage to the Myocardium Caused by Cocaine Administration

**Figure 5:**
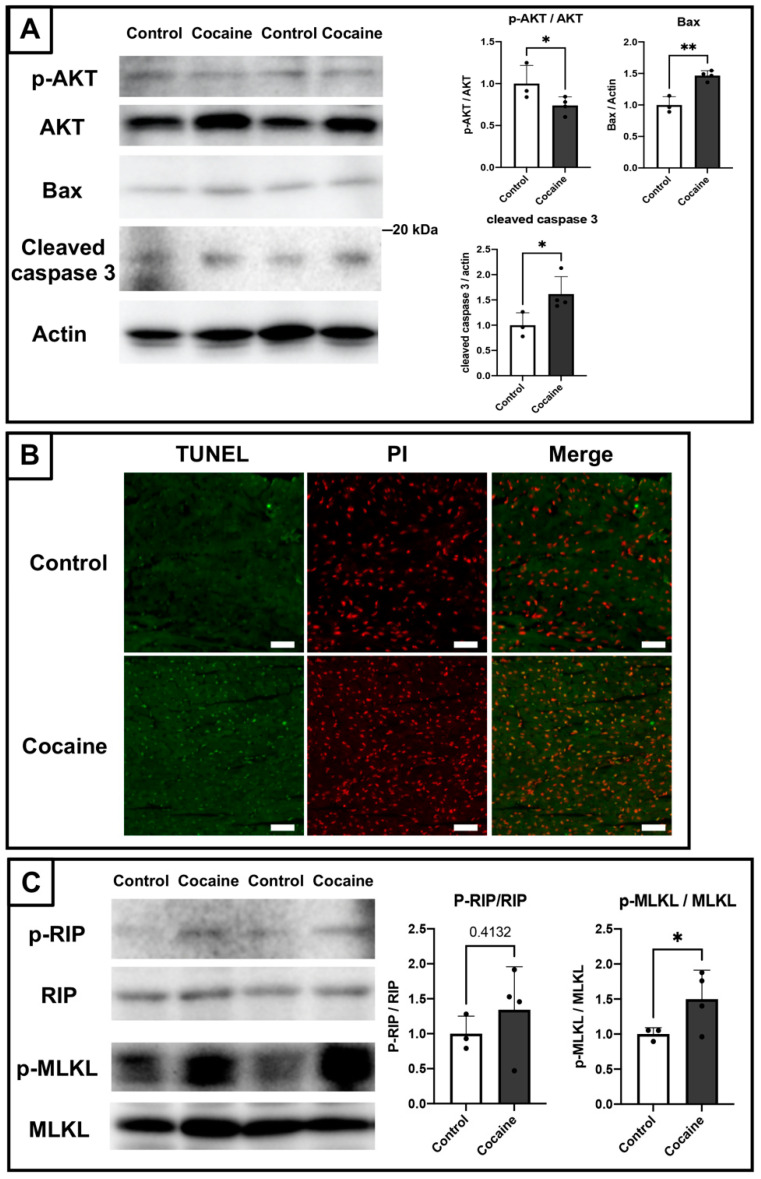
Occurrence of apoptosis and incomplete necroptosis in the myocardium after 14 days of cocaine administration. (**A**) Protein expressions of protein kinase B (AKT), phosphorylated AKT at ser473 (p-AKT), BCL2 Associated X (BAX), and cleaved caspase 3 in left ventricles from the control and cocaine groups. Levels of actin served as the internal control. (**B**) TUNEL staining in the myocardium. Nuclei were counterstained with propidium iodide (PI). Scale bars = 250 μm. (**C**) Protein expressions of receptor-interacting protein (RIP), phosphorylated RIP at ser166 (p-RIP), mixed lineage kinase domain-like protein (MLKL), and phosphorylated MLKL (p-MLKL) in left ventricles from the control and cocaine groups. Levels of actin served as the internal control. Individual values are presented in black bullets. Each bar represents mean and S.D. *, *p* < 0.05, **, *p* < 0.01.

As mentioned in “Introduction”, dephosphorylation of cardiac Cx43 leads to the apoptosis of cardiomyocytes. We therefore examined the expressions of apoptosis-related markers by immunoblotting. Protein kinase B (Akt) is an important intracellular regulator that inhibits apoptosis [37]. We found a downregulation of the proportion of the phosphorylated (activated) form of Akt at serine 473 in total Akt, along with increased expressions of the proapoptotic protein BCL2 associated X (Bax), and the activated apoptotic marker cleaved caspase-3 [38] in the myocardium of the cocaine group as compared to the control group (Figure 5A). Furthermore, terminal deoxynucleotidyl transferase dUTP nick end labeling (TUNEL) analysis also revealed apparently increased TUNEL-positive stained cardiomyocytes in the cocaine group (Figure 5B). These findings indicate the occurrence of apoptosis in the myocardium after 14 days of cocaine administration via the tail vein.

In addition to apoptosis, there are many other pathways implicated in the damage to the myocardium. For example, Ca^2+^ overload and oxidative stress can lead to cardiomyocyte death by either apoptotic or necrotic pathways [6]. Therefore, we also explored whether there are other cell death pathways active in the myocardium of the cocaine group, such as, for example, caspase-independent necroptosis [39]. Immunoblotting of necroptosis-related mediators revealed a significantly activated mixed lineage kinase domain-like protein (MLKL) yet only a slightly activated receptor-interacting protein (RIP) that was not statistically significant (Figure 5C). Thus, in addition to apoptosis, the necroptosis machinery might also play some role in the damage to the myocardium caused by cocaine administration. Considering that necroptosis plays a more important role in inflammation than apoptosis, the incomplete development of necroptosis seems to be consistent with the limited inflammatory findings in the myocardium of the cocaine group (Figure 1).

### 2.6. Possible Involvement of Adiponectin in Protecting the Myocardium after Cocaine Administration

**Figure 6:**
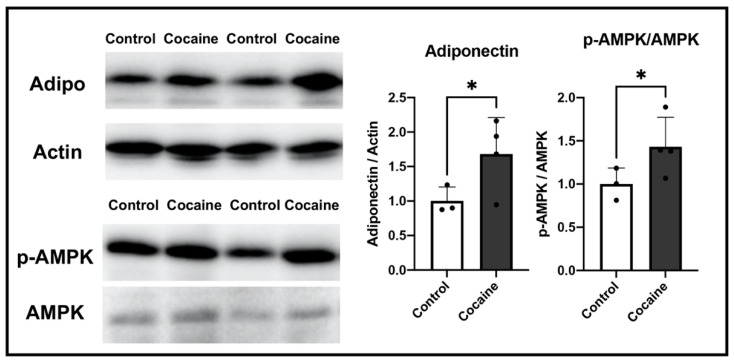
Possible involvement of adiponectin in the myocardium after 14 days of cocaine administration. Protein expressions of adiponectin (Adipo), AMP-activated protein kinase (AMPK), and phosphorylated AMPK (p-AMPK) in left ventricles from the control and cocaine groups. Levels of actin served as the internal control. Individual values are presented in black bullets. Each bar represents mean and S.D. *, *p* < 0.05.

In order to obtain further insight into the changes in cellular status in the myocardium after cocaine treatment, we conducted a DNA microarray analysis. Table 1 lists the 10 genes that were most often upregulated in response to cocaine treatment. The microarray results revealed adiponectin (*Adipoq*) to be the most upregulated gene in the myocardium of the cocaine group (Table 1). Adiponectin has been shown to accumulate in the myocardium in response to stress or injury. As a result, we performed immunoblotting in order to examine the expressions of adiponectin and its important effector AMP-activated protein kinase (AMPK). The levels of both proteins were significantly increased in the cocaine group (Figure 6), indicating the possible role of adiponectin in protecting the myocardium against cocaine toxicity. In the myocardium, adiponectin is mainly secreted by epicardial adipose tissue, with relatively limited amounts secreted by cardiomyocytes [40,41]. Our findings suggest that the increased level of adiponectin in the myocardium after 14 days of cocaine administration is mainly derived either from the epicardium or systematically, rather than from cardiomyocytes.

## 3. Discussion

Although CBN has been repeatedly reported among the postmortem findings in the myocardium of cocaine abusers, it is rarely reported in animal experiments of cocaine cardiotoxicity. In previous studies, CBN was induced separately by catecholamines or ischemia/reperfusion injury [10]. Ischemia/reperfusion-related CBN is associated with massive interstitial hemorrhaging with subsequent inflammatory infiltration [11]. In contrast, inflammation is not an inevitable event in catecholamine-induced CBN [42]. In our experiments, massive interstitial hemorrhaging and inflammation were not found in the myocardium after 14 days of cocaine administration via the tail vein. This suggests that cocaine-induced CBN is more likely attributable to catecholamine overload. Similarly, the presence of CBN and the absence of inflammatory infiltration have also been reported in heart samples from cocaine abusers [43]. The sparsely observed eosinophil-dominated myocarditis in the cocaine group is consistent with the characteristics of hypersensitivity myocarditis. Although not commonly seen, there are reports in which eosinophilic myocarditis was confirmed in cocaine dependents [1,43]. These might be related to the hypersensitive reaction towards cocaine or its metabolites. It is worth noting that intracellular calcium assay for cardiomyocytes will be helpful for better elucidation of the current findings, and is recommended for interested researchers.

Due to the short half-life of Cx43, most studies have been conducted in vivo or ex vivo, wherein the heart was harvested immediately (mostly less than 60 min) after pre-conditioning, in order to determine the instant status of Cx43 [15,44]. Although the hearts were harvested 24 h after the last administration in our experiments, we found an increased dephosphorylation of Cx43 under stress and injury that is consistent with previous findings [16]. This might reflect the persistent effect of cocaine or the relatively long-term cocaine administration in our experiments. It has been reported that myocardial damage induced by ischemia/reperfusion or diseases results in the rearrangement of Cx43, and is closely related to myocardial function [45]. Though it is fascinating to explore whether cocaine has a similar effect on cardiac Cx43, the rearrangement of Cx43 was not observed in our chronic cocaine administrated study. An acute administration model, either in vivo or ex vivo with instant sampling, may be more applicable to this point. There is evidence to suggest that the dephosphorylation of Cx43 contributes to arrhythmias and further cardiac injury [46]. In the myocardium of the cocaine group, we found increased intercalated, disc-localized, dephosphorylated Cx43 in CBN adjacent regions, which may play a role in cocaine-induced cardiac disorders. The current preliminary findings can serve as the basis of therapeutic strategies against cardiac consequences in cocaine addicts. Further examination of cardiac function will be beneficial for advancing our understanding of this point. It should also be noted that there are different phosphorylation sites of Cx43, whereas only serine 368 was determined in our study. Considering the interesting increased expression of total Cx43, the status of other phosphorylation sites of Cx43 would be worth pursuing in subsequent explorations. Aside from its canonical role, Cx43 is also detected in mitochondria, where it is implicated in processes including respiration and ROS generation [47]. The link between cocaine-induced mitochondrial damage and the role of mitochondrial Cx43 is another topic which requires illumination [48]. Co-immunoprecipitation assay or immunoelectron microscopy should be appropriate techniques in this field [49].

Our DNA microarray-based attempt to seek gene expression alterations after cocaine administration resulted in the unexpected finding that adiponectin is the gene most affected by cocaine under our experimental conditions. The cardiac protective role of adiponectin-AMPK has been well established in various models, including cardiac ischemia/reperfusion injury, diabetes, and cardiac hypertrophy [50,51,52]. It has been reported that cocaine exposure increases systematic adiponectin levels [53]. In our previous study on short-term cocaine administration, mitochondrial fission was found to act as an early compensation mechanism against the cardiotoxicity of cocaine, prior to the occurrence of structural changes [54]. The beneficial effect of adiponectin is also considered an early event in cardiac pathogenesis [28]. Combined with our current findings, adiponectin may act protectively against cocaine cardiotoxicity during the early stages of cardiac pathogenesis. It is worth noticing that high molecular weight (multimeric) adiponectin is assumed to be the biologically active form [55], whereas the adiponectin observed in our experiments consisted of both multimeric and monomeric forms. This may limit our ability to interpret the increased adiponectin level within the heart in this study. An in-depth elucidation of the role of adiponectin in protecting against the cardiotoxicity of cocaine requires further study, for instance, by applying knockout mice or antagonists of adiponectin-related pathways. It should be noted that the limitation of this study is the relatively small sample size; a follow-up study based on a bigger population is being pursued.

In conclusion, we have demonstrated that the administration of 20 mg/kg/day cocaine to rats through the tail vein for 14 days results in disordered myocardium with widespread CBN, mitochondrial dysfunction, increased dephosphorylation of Cx43, involvement of apoptosis and necroptosis, and participation of cardiac protective adiponectin. Differences among individual models may be present; yet, our findings should be helpful for advancing understanding of the cardiotoxicity of relatively long-term exposure to cocaine, as well as establishing novel therapeutic strategies in this field.

## 4. Materials and Methods

All methods were carried out in accordance with relevant guidelines and regulations.

### 4.1. Animals and Cocaine Administration

Animal experiments were approved by the Institutional Animal Care and Use Committee of Tokyo Medical and Dental University, and in keeping with Animal Research: Reporting of In Vivo Experiments (ARRIVE) guidelines and regulations. Eight-week-old male Sprague Dawley rats were randomly divided into 2 groups and administered saline (control group, *n* = 3) or cocaine (cocaine group, *n* = 4). The dose of cocaine was 20 mg/kg/day after dose conversions from humans to rats, in order to reproduce the typical serum concentrations approaching acute cocaine poisoning overdose in humans [56,57]. Cocaine hydrochloride (CAS: 50-36-2, Shionogi & Co., Ltd., Osaka, Japan) was dissolved in saline and administered intravenously through the tail vein once a day for 14 days. The same volume of saline was given via the tail vein for control group rats on the same schedule. Twenty-four hours after the last administration, the rats were sacrificed by intraperitoneal injection of an overdose of sodium pentobarbital (40 mg/kg). The hearts were collected immediately and placed in phosphate-buffered saline solution (PBS, 4 °C, pH 7.4). The left ventricles were then evenly separated for the following histological and biochemical analyses.

### 4.2. Histological Analysis and Immunohistochemical Staining

Sections of the left ventricular specimens (2.5 μm thickness) for microscopy were prepared from tissues fixed in 4% paraformaldehyde and embedded in paraffin. Hematoxylin-eosin (H&E), Elastica Masson-Goldner (EMG), and Phosphotungstic Acid Hematoxylin (PTAH) staining were conducted for histological analysis of the myocardium. Immunohistochemical staining of dephosphorylated Cx43 was performed. In brief, deparaffinized sections were heated for 5 min in a 10 mM citrate buffer (pH 6.0) for antigen retrieval. Next, the activity of endogenous peroxidase was quenched by applying 0.3% hydrogen peroxide. The sections were then incubated with mouse 8-OHdG monoclonal antibody (diluted 100-fold; JaICA, Shizuoka, Japan) or mouse Cx43 monoclonal antibody (diluted 100-fold; #13-8300, Thermo Fisher Scientific, Waltham, MA, USA) overnight at 4 °C. The following visualization of antigens was conducted applying Histofine Simple Stain MAX-PO (Multi) (Nichirei Biosciences Inc., Tokyo, Japan) and diaminobenzidine (Nichirei Biosciences Inc.) as the substrate. Controls for the immunostaining procedures were prepared by incubation in PBS in place of the primary antibody.

### 4.3. Transmission Electron Microscopy

Left ventricle specimens for transmission electron microscopy were prepared by washing the tissues in 0.1 M phosphate buffer (PB) and then fixing them with 2.5% glutaraldehyde and 4.5% paraformaldehyde in PB. The fixed specimens were then dehydrated and embedded in Epon epoxy resin after incubation in 1% osmium tetroxide for 2 h. Subsequently, ultrathin sections were prepared and stained with uranyl acetate and lead citrate. The sections were observed under a transmission electron microscope (H7100, Hitachi, Japan) and recorded using an AMT Advantage-HS CCD camera (AMT, Woburn, MA, USA). The number of mitochondria per field (magnitude at 10,000×) was recorded from 10 electron micrographs in each group. Cross-sectional areas of randomly selected mitochondria (*n* = 100 for each group) in longitude views of cardiac myofibrils at fields magnitude at 10,000× were counted using ImageJ software (ver1.53a).

### 4.4. Mitochondrial Electron Transport Chain Activity Detection

Left ventricle specimens for enzyme activity assays were immediately frozen at −80 °C. A Mitochondria Isolation Kit (ab110168, Abcam, Cambridge, UK) was used to extract mitochondria from the left ventricle. The protein concentrations of the extracts were determined and adjusted to 100 μg/mL by adding PBS. Mitochondrial oxidative phosphorylation (OXPHOS) Complex I enzyme activity was determined using a Complex I Enzyme Activity Assay Kit (ab109721, Abcam).

### 4.5. Immunoblotting

The lysates of left ventricle samples for immunoblotting were prepared in an STE buffer (320 mM sucrose, 10 mM Tris-HCl, 5 mM EDTA, 50 mM NaF, 2 mM Na_3_VO_4_, protease inhibitor cocktail (Roche Diagnostics, Mannheim, Germany)) for subsequent SDS-polyacrylamide gel electrophoresis. Next, immunoblotting was performed with total OXPHOS Rodent WB antibody cocktail (ab110413, Abcam), translocase of the outer mitochondrial membrane 20 (TOM20) antibody (#42406, Cell Signal Technology, Beverly, MA, USA), anti-4-hydroxy-2-nonenal (4-HNE) antibody (MHN-100P, Japan Institute for the Control of Aging (JaICA), Nikken SEIL Co., Ltd., Shizuoka, Japan), total Cx43 antibody (MAB3067, EMD Millipore Corporation, Burlington, CA, USA), and dephosphorylated Cx43 antibody (Thermo Fisher Scientific). Cx43 antibodies were selected based on a former study [21], phosphorylated protein kinase B (Akt, Ser473, phosphorylation site was selected according to former study [37]) antibody (#9271, Cell Signal Technology), Akt antibody (#9272, Cell Signal Technology), BCL2 Associated X (Bax) antibody (#5023, Cell Signal Technology), cleaved caspase-3 antibody (#9661, Cell Signal Technology), phosphorylated-receptor-interacting protein (RIP, Ser166) antibody (#44590, Cell Signal Technology), total RIP antibody (#551041, BD Biosciences, USA), phosphorylated-mixed lineage kinase domain-like protein (MLKL, Ser358) antibody (#17-10400, EMD Millipore Corporation, Burlington, CA, USA), MLKL antibody (ab189612, Abcam), adiponectin antibody (GTX112777, GeneTex, Inc., Irvine, CA, USA), phosphorylated-AMP-activated protein kinase (AMPK, Thr172) antibody (#2535, Cell Signal Technology), total AMPK antibody (#5831, Cell Signal Technology), and actin antibody (A2066, Sigma-Aldrich, St. Louis, MO, USA). Visualization of antigens was conducted with peroxidase-bonded anti-mouse or rabbit IgG secondary antibodies (Promega Corporation, Madison, WI, USA) and enhanced chemiluminescence reagents (Thermo Fisher Scientific). The quantification of band densities was performed using CS analyzer 4 image analyzing software (Atto, Tokyo, Japan).

### 4.6. Real-Time Reverse Transcriptase-Mediated PCR Analysis

Real-time reverse transcriptase-mediated PCR analysis was performed as previously described [58]. The primers used are listed in Supplementary Table S1.

### 4.7. TUNEL Analysis

TUNEL analysis was performed using MEBSTAIN Apoptosis TUNEL Kit Direct (MLB International Corporation, New York, NY, USA). FITC and RHOD fluorescence were observed under a fluorescence microscope (DMi8, Leica).

### 4.8. Transcriptome Analysis

The total RNA of rats’ left ventricle samples for transcriptome analysis was extracted using a TRIzol reagent (Thermo Fisher Scientific) and further purified using a RNeasy Mini Kit (Qiagen, MD, USA). The purified total RNA was examined for integrity on a BioAnalyzer (Agilent Technologies) and hybridized into ClariomS array (Thermo Fisher Scientific). The results were deposited in the GEO database (https://www.ncbi.nlm.nih.gov/geo/, accession number GSE200127, accessed on 7 April 2022). Microarray Data Analysis was conducted using Transcriptome Analysis Console (TAC) software (Thermo Fisher Scientific).

### 4.9. Statistical Analysis

Student’s t-test was applied in order to assess statistical significance. *p* < 0.05 was considered significant. Statistical analysis was conducted using GraphPad Prism (Version 9.0.0, GraphPad Software, San Diego, CA, USA).

## Figures and Tables

**Table 1:** Rats’ left ventricular genes upregulated by cocaine.

	Gene	Fold Change	Description
1	*Adipoq*	14.28	adiponectin
2	*Ttn*	9.55	titin
3	*Car3*	6.1	Carbonic anhydrase 3
4	*Tnnt2*	5.51	Troponin T type 2
5	*Mb*	5.39	myoglobin
6	*Alox15*	4.92	Arachidonate 15-lipoxygenase
7	*Abcb1b*	4.57	ATP-binding cassette, subfamily B, member 1B
8	*Myh6*	4.19	Myosin heavy chain 6
9	*Myl7*	3.83	Myosin light chain 7
10	*Lcn2*	3.63	lipocalin2

Top 10 upregulated genes in the left ventricle in order of fold change (cocaine group compared to control group).

## Data Availability

The authors confirm that the data supporting the findings of this study are available within the article and its Supplementary Materials.

## Supplementary Materials

The supporting information can be downloaded at: https://www.mdpi.com/article/10.3390/ijms231911978/s1.

## References

[1] Isner J.M., Estes N.M., Thompson P.D., Costanzo-Nordin M.R., Subramanian R., Miller G., Katsas G., Sweeney K., Sturner W.Q. (1986). Acute Cardiac Events Temporally Related to Cocaine Abuse. N. Engl. J. Med..

[2] Kloner R.A., Hale S., Alker K., Rezkalla S. (1992). The Effects of Acute and Chronic Cocaine Use on the Heart. Circulation.

[3] Phillips K., Luk A., Soor G.S., Abraham J.R., Leong S., Butany J. (2009). Cocaine Cardiotoxicity: A Review of the Pathophysiology, Pathology, and Treatment Options. Am. J. Cardiovasc. Drugs.

[4] Crumb W.J., Clarkson C.W. (1990). Characterization of Cocaine-Induced Block of Cardiac Sodium Channels. Biophys. J..

[5] Pitts W.R., Lange R.A., Cigarroa J.E., Hillis L.D. (1997). Cocaine-Induced Myocardial Ischemia and Infarction: Pathophysiology, Recognition, and Management. Prog. Cardiovasc. Dis..

[6] Georgieva E., Karamalakova Y., Miteva R., Abrashev H., Nikolova G. (2021). Oxidative Stress and Cocaine Intoxication as Start Points in the Pathology of Cocaine-Induced Cardiotoxicity. Toxics.

[7] Fineschi V., Wetli C.V., Paolo M.D., Baroldi G. (1997). Myocardial Necrosis and Cocaine. A Quantitative Morphologic Study in 26 Cocaine-Associated Deaths. Int. J. Leg. Med..

[8] Tazelaar H.D., Karch S.B., Stephens B.G., Billingham M.E. (1987). Cocaine and the Heart. Hum. Pathol..

[9] Karch S.B., Billingham M.E. (1986). Myocardial Contraction Bands Revisited. Hum. Pathol..

[10] Todd G.L., Baroldi G., Pieper G.M., Clayton F.C., Eliot R.S. (1985). Experimental Catecholamine-Induced Myocardial Necrosis. I. Morphology, Quantification and Regional Distribution of Acute Contraction Band Lesions. J. Mol. Cell Cardiol..

[11] Baroldi G. (1975). Different Types of Myocardial Necrosis in Coronary Heart Disease: A Pathophysiologic Review of Their Functional Significance. Am. Heart J..

[12] Karch S.B. (1987). Resuscitation-Induced Myocardial Necrosis. Catecholamines and Defibrillation. Am. J. Forensic. Med. Pathol..

[13] Garcia-Dorado D., Inserte J., Ruiz-Meana M., González M.A., Solares J., Juliá M., Barrabes J.A., Soler-Soler J. (1997). Gap Junction Uncoupler Heptanol Prevents Cell-to-Cell Progression of Hypercontracture and Limits Necrosis During Myocardial Reperfusion. Circulation.

[14] Ruiz-Meana M., Garcia-Dorado D., Hofstaetter B., Piper H.M., Soler-Soler J. (1999). Propagation of Cardiomyocyte Hypercontracture by Passage of Na(+) through Gap Junctions. Circ. Res..

[15] Shintani-Ishida K., Unuma K., Yoshida K. (2009). Ischemia Enhances Translocation of Connexin43 and Gap Junction Intercellular Communication, Thereby Propagating Contraction Band Necrosis after Reperfusion. Circ. J..

[16] Severs N.J., Bruce A.F., Dupont E., Rothery S. (2008). Remodelling of Gap Junctions and Connexin Expression in Diseased Myocardium. Cardiovasc. Res..

[17] Schulz R., Görge P.M., Görbe A., Ferdinandy P., Lampe P.D., Leybaert L. (2015). Connexin 43 Is an Emerging Therapeutic Target in Ischemia/Reperfusion Injury, Cardioprotection and Neuroprotection. Pharmacol. Ther..

[18] Lampe P.D., Cooper C.D., King T.J., Burt J.M. (2006). Analysis of Connexin43 Phosphorylated at S325, S328 and S330 in Normoxic and Ischemic Heart. J. Cell Sci..

[19] Solan J.L., Marquez-Rosado L., Sorgen P.L., Thornton P.J., Gafken P.R., Lampe P.D. (2007). Phosphorylation at S365 Is a Gatekeeper Event That Changes the Structure of Cx43 and Prevents Down-Regulation by Pkc. J. Cell Biol..

[20] Xiao S., Shimura D., Baum R., Hernandez D.M., Agvanian S., Nagaoka Y., Katsumata M., Lampe P.D., Kleber A.G., Hong T. (2020). Auxiliary Trafficking Subunit Gja1-20k Protects Connexin-43 from Degradation and Limits Ventricular Arrhythmias. J. Clin. Invest..

[21] Beardslee M.A., Lerner D.L., Tadros P.N., Laing J.G., Beyer K.A., Yamada A., Kleber G., Schuessler R.B., Saffitz J.E. (2000). Dephosphorylation and Intracellular Redistribution of Ventricular Connexin43 During Electrical Uncoupling Induced by Ischemia. Circ. Res..

[22] Yang Y., Yan X., Xue J., Zheng Y., Chen M., Sun Z., Liu T., Wang C., You H., Luo D. (2019). Connexin43 Dephosphorylation at Serine 282 Is Associated with Connexin43-Mediated Cardiomyocyte Apoptosis. Cell Death Differ..

[23] Lo C.W. (2000). Role of Gap Junctions in Cardiac Conduction and Development: Insights from the Connexin Knockout Mice. Circ Res..

[24] Saffitz J.E. (2009). Arrhythmogenic Cardiomyopathy and Abnormalities of Cell-to-Cell Coupling. Heart Rhythm.

[25] Hui X., Lam K.S., Vanhoutte P.M., Xu A. (2012). Adiponectin and Cardiovascular Health: An Update. Br. J. Pharmacol..

[26] Yavuz S., Ece A. (2014). Mean Platelet Volume as an Indicator of Disease Activity in Juvenile Sle. Clin. Rheumatol..

[27] Fang H., Judd R.L. (2018). Adiponectin Regulation and Function. Compr. Physiol..

[28] Caselli C., D’Amico A., Cabiati M., Prescimone T., Del Ry S., Giannessi D. (2014). Back to the heart: The protective role of adiponectin. Pharmacol. Res..

[29] Leffler K.E., Abdel-Rahman A.A. (2020). Restoration of Adiponectin-Connexin43 Signaling Mitigates Myocardial Inflammation and Dysfunction in Diabetic Female Rats. J. Cardiovasc. Pharmacol..

[30] Liu L., Yan M., Yang R., Qin X., Chen L., Li L., Si J., Li X., Ma K. (2021). Adiponectin Attenuates Lipopolysaccharide-Induced Apoptosis by Regulating the Cx43/Pi3k/Akt Pathway. Front. Pharmacol..

[31] Moritz F., Monteil C., Isabelle M., Bauer F., Renet S., Mulder P., Richard V., Thuillez C. (2003). Role of Reactive Oxygen Species in Cocaine-Induced Cardiac Dysfunction. Cardiovasc. Res..

[32] Fineschi V., Baroldi G., Centini F., Cerretani D., Fiaschi A.I., Micheli L., Parolini M., Turillazzi E., Giorgi G. (2001). Markers of Cardiac Oxidative Stress and Altered Morphology after Intraperitoneal Cocaine Injection in a Rat Model. Int. J. Leg. Med..

[33] Todd G.L., Cullan G.E., Cullan G.M. (1980). Isoproterenol-Induced Myocardial Necrosis and Membrane Permeability Alterations in the Isolated Perfused Rabbit Heart. Exp. Mol. Pathol..

[34] Srinivasan S., Avadhani N.G. (2012). Cytochrome C Oxidase Dysfunction in Oxidative Stress. Free Radic. Biol. Med..

[35] Liu Y., Fiskum G., Schubert D. (2002). Generation of reactive oxygen species by the mitochondrial electron transport chain. J. Neurochem..

[36] Peng S.K., French W.J., Pelikan P.C. (1989). Direct cocaine cardiotoxicity demonstrated by endomyocardial biopsy. Arch. Pathol. Lab. Med..

[37] Zhou H., Li X.-M., Meinkoth J., Pittman R.N. (2000). Akt Regulates Cell Survival and Apoptosis at a Postmitochondrial Level. J. Cell Biol..

[38] Pawlowski J., Kraft A.S. (2000). Bax-induced apoptotic cell death. Proc. Natl. Acad. Sci. USA.

[39] Silke J., Rickard J.A., Gerlic M. (2015). The Diverse Role of Rip Kinases in Necroptosis and Inflammation. Nat. Immunol..

[40] Hirata Y., Kurobe H., Akaike M., Chikugo F., Hori T., Bando Y., Nishio C., Higashida M., Nakaya Y., Kitagawa T. (2011). Enhanced Inflammation in Epicardial Fat in Patients with Coronary Artery Disease. Int. Heart J..

[41] Piñeiro R., Iglesias M.J., Gallego R., Raghay K., Eiras S., Rubio J., Diéguez C., Gualillo O., Gonzalez-Juanatey J.R., Lago F. (2005). Adiponectin is synthesized and secreted by human and murine cardiomyocytes. FEBS Lett..

[42] Rosenbaum J.S., Billingham M.E., Ginsburg R., Tsujimoto G., Lurie K.G., Hoffman B.B. (1988). Cardiomyopathy in a Rat Model of Pheochromocytoma. Morphological and Functional Alterations. Am. J. Cardiovasc. Pathol..

[43] Virmani R., Robinowitz M., Smialek J.E., Smyth D.F. (1988). Cardiovascular Effects of Cocaine: An Autopsy Study of 40 Patients. Am. Heart J..

[44] Beardslee M.A., Laing J.G., Beyer E.C., Saffitz J.E. (1998). Rapid Turnover of Connexin43 in the Adult Rat Heart. Circ. Res..

[45] Görbe A., Varga Z.V., Kupai K., Bencsik P., Kocsis G.F., Csont T., Boengler K., Schulz R., Ferdinandy P. (2011). Cholesterol diet leads to attenuation of ischemic preconditioning-induced cardiac protection: The role of connexin 43. Am. J. Physiol. Circ. Physiol..

[46] Solan J.L., Lampe P.D. (2009). Connexin43 phosphorylation: Structural changes and biological effects. Biochem. J..

[47] Martins-Marques T., Ribeiro-Rodrigues T.M., Batista-Almeida D., Aasen T., Kwak B.R., Girao H. (2019). Biological Functions of Connexin43 Beyond Intercellular Communication. Trends Cell Biol..

[48] Varga Z.V., Ferdinandy P., Liaudet L., Pacher P. (2015). Drug-induced mitochondrial dysfunction and cardiotoxicity. Am. J. Physiol. Circ. Physiol..

[49] Wang M., Smith K., Yu Q., Miller C., Singh K., Sen C.K. (2019). Mitochondrial connexin 43 in sex-dependent myocardial responses and estrogen-mediated cardiac protection following acute ischemia/reperfusion injury. Basic Res. Cardiol..

[50] Shibata R., Sato K., Pimentel D.R., Takemura Y., Kihara S., Ohashi K., Funahashi T., Ouchi N., Walsh K. (2005). Adiponectin protects against myocardial ischemia-reperfusion injury through AMPK- and COX-2–dependent mechanisms. Nat. Med..

[51] Essick E.E., Ouchi N., Wilson R.M., Ohashi K., Ghobrial J., Shibata R., Pimentel D.R., Sam F. (2011). Adiponectin mediates cardioprotection in oxidative stress-induced cardiac myocyte remodeling. Am. J. Physiol. Circ. Physiol..

[52] Shibata R., Ouchi N., Ito M., Kihara S., Shiojima I., Pimentel D.R., Kumada M., Sato K., Schiekofer S., Ohashi K. (2004). Adiponectin-mediated modulation of hypertrophic signals in the heart. Nat. Med..

[53] You Z.B., Wang B., Gardner E.L., Wise R.A. (2019). Cocaine and Cocaine Expectancy Increase Growth Hormone, Ghrelin, Glp-1, Igf-1, Adiponectin, and Corticosterone While Decreasing Leptin, Insulin, Gip, and Prolactin. Pharmacol. Biochem. Behav..

[54] Wen S., Unuma K., Funakoshi T., Aki T., Uemura K. (2021). Altered cardiac mitochondrial dynamics and biogenesis in rat after short-term cocaine administration. Sci. Rep..

[55] Aso Y., Yamamoto R., Wakabayashi S., Uchida T., Takayanagi K., Takebayashi K., Okuno T., Inoue T., Node K., Tobe T. (2006). Comparison of Serum High-Molecular Weight (Hmw) Adiponectin with Total Adiponectin Concentrations in Type 2 Diabetic Patients with Coronary Artery Disease Using a Novel Enzyme-Linked Immunosorbent Assay to Detect Hmw Adiponectin. Diabetes.

[56] Heard K., Palmer R., Zahniser N.R. (2008). Mechanisms of Acute Cocaine Toxicity. Open Pharmacol. J..

[57] Nair A.B., Jacob S. (2016). A simple practice guide for dose conversion between animals and human. J. Basic Clin. Pharm..

[58] Funakoshi T., Furukawa M., Aki T., Uemura K. (2019). Repeated exposure of cocaine alters mitochondrial dynamics in mouse neuroblastoma Neuro2a. NeuroToxicology.

