# Supplementary material for: Contraction Band Necrosis with Dephosphorylated Connexin 43 in Rat Myocardium after Daily Cocaine Administration
Source: Int J Mol Sci. 2022 Oct 9;23(19):11978. doi: 10.3390/ijms231911978 (PMC9570416; doi:10.3390/ijms231911978)
Supplement: Supplementary file 1 [file ijms-23-11978-s001.zip › ijms-1902224-supplementary.pdf]

Supplementary Information

Contraction band necrosis with dephosphorylated connexin 43 in rat myocardium after daily cocaine administration

Shuheng Wen[1], Kana Unuma[1], Takeshi Funakoshi[1], Toshihiko Aki[1], and Koichi Uemura[1]

[1] Department of Forensic Medicine, Graduate School of Medical and Dental Sciences, Tokyo Medical and Dental University, Tokyo, Japan

Supplementary Table S1. qPCR Primer Sequences

Gene	Primer	
mt-Co1 (rat)	Forward (5'-3')	GCT TTT GAC TGC TTC CTC CA
	Reverse (3'-5')	GCT AGG TTT CCG GCT AAG GG
mt-Co2 (rat)	Forward (5'-3')	ACA AGA CGC CAC ATC ACC TA
	Reverse (3'-5')	TGG GCG TCT ATT GTG CTT GT
mt-Co3 (rat)	Forward (5'-3')	AAA GGC CTC CGA TAC GGA AT
	Reverse (3'-5')	AAT TCC TGT TGG GGG TCA GC
Cox4i1 (rat)	Forward (5'-3')	TCT ACT TCG GTG TGC CTT CG
	Reverse (3'-5')	CCA CAT CAG GCA AGG GGT AG
Cox5a (rat)	Forward (5'-3')	TTT GAT GCT CGC TGG GTG AC
	Reverse (3'-5')	ACA TGC CCT CAA AGC AGC AT
Cox6a1 (rat)	Forward (5'-3')	TGA AGT CGC GAC ACG AAG AG
	Reverse (3'-5')	TTC ATA GCC AGT CGG AAG CG
Cox6a2 (rat)	Forward (5'-3')	CTA TCA CCA CCT CCG CAT CC
	Reverse (3'-5')	GAG TCT TCA AGG CTG CTC GT

mt-Co1: cytochrome c oxidase subunit I, mt-Co2: cytochrome c oxidase subunit II, mt-Co3: cytochrome c oxidase subunit III, Cox4i1: cytochrome c oxidase subunit 4I1, Cox5a: cytochrome c oxidase subunit 5A, Cox6a1: cytochrome c oxidase subunit 6A1, Cox6a2: cytochrome c oxidase subunit 6A2.

Supplementary Figure S1

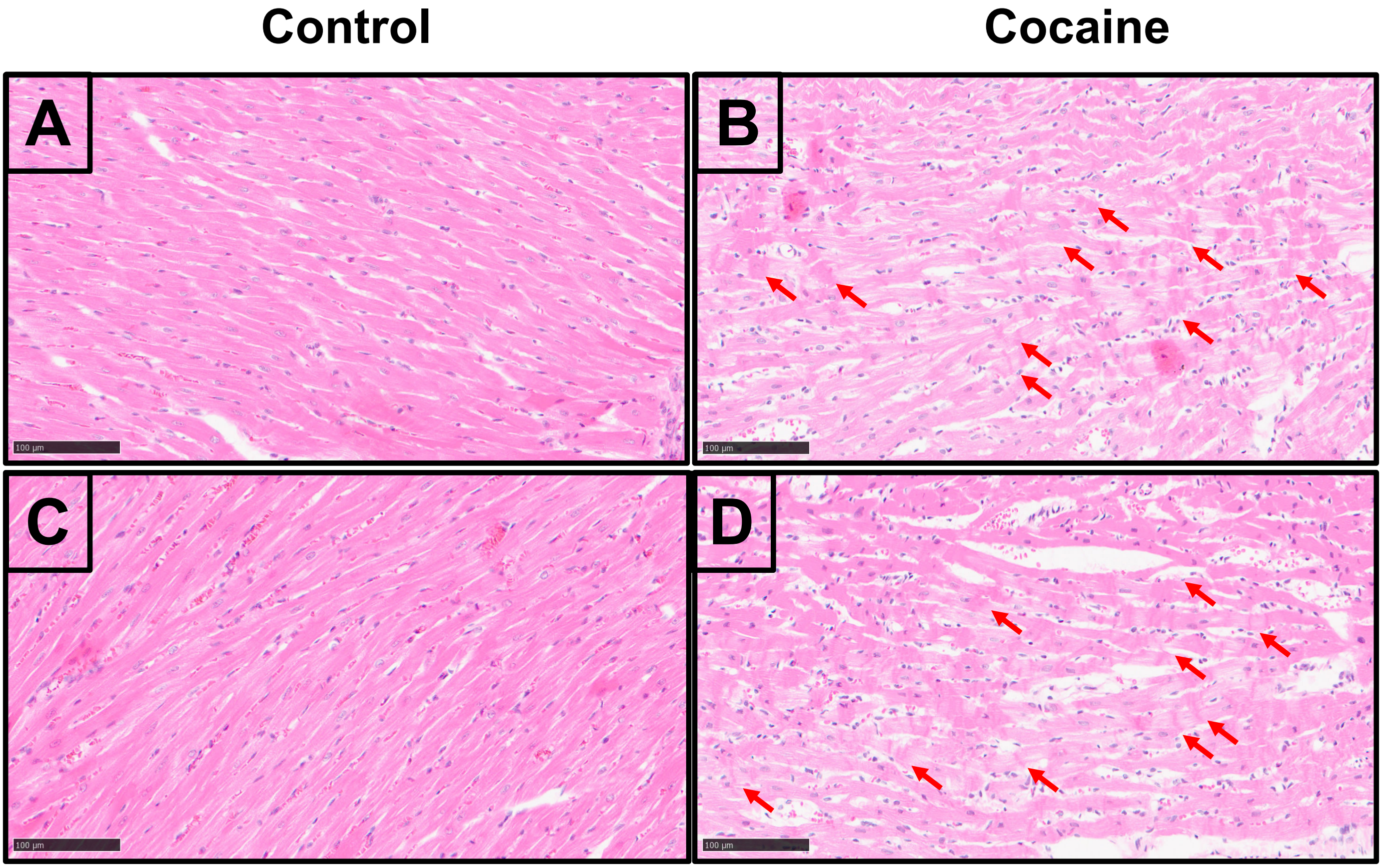

Hematoxylin and Eosin staining of myocardium after 14 days of cocaine administration. Left ventricular from (**A**), (**C**) control, and (**B**), (**D**) cocaine group indicating widespread CBN-like hypereosinophilic transverse bands in myocardium from the cocaine group (arrowheads). scale bars = 100 µm.
